# CRISPR as a Tool to Uncover Gene Function in Polycystic Ovary Syndrome: A Literature Review of Experimental Models Targeting Ovarian and Metabolic Genes

**DOI:** 10.3390/cells14221769

**Published:** 2025-11-12

**Authors:** Shahd Bucheeri, Yasmine Alcibahy, Yara Bucheeri, Sarah Bucheeri, Abrar Alhermi, Alexandra E. Butler

**Affiliations:** 1 School of Medicine, Royal College of Surgeons in Ireland, Medical University of Bahrain, Busaiteen P.O. Box 15503, Bahrain; 21200330@rcsi.com (S.B.); 21201313@rcsi.com (Y.A.); 24200314@rcsi.com (Y.B.); 24200120@rcsi.com (S.B.); 20205606@rcsi.com (A.A.); 2 Research Department, Royal College of Surgeons in Ireland, Medical University of Bahrain, Busaiteen P.O. Box 15503, Bahrain

**Keywords:** polycystic ovary syndrome (PCOS), CRISPR/Cas9, gene editing, functional genomics

## Abstract

**Highlights:**

**What are the main findings?**

1. CRISPR models reveal gene-specific contributions to ovarian and metabolic PCOS phenotypes, including hyperandrogenism and insulin resistance.
2. Single-gene models show species-specific effects, highlighting the need for dual-gene and integrated models.

**What are the implications of the main findings?**

1. CRISPR studies identify potential diagnostic markers and clarify gene-hormone interactions in PCOS.
2. Combined gene editing and hormonal models provide a platform for studying the polygenic, multifactorial nature of PCOS.

**Abstract:**

Polycystic ovary syndrome (PCOS) is a complex disorder characterized by reproductive abnormalities such as hyperandrogenism, ovulatory dysfunction, and polycystic ovarian morphology, and is frequently accompanied by metabolic disturbances such as insulin resistance, obesity and dyslipidemia. Genome-wide association studies (GWASs) have identified several susceptibility loci, yet little is known about their functional implications. Clustered regularly interspaced short palindromic repeats (CRISPR)/CRISPR-associated protein 9 (CRISPR/Cas9) has emerged as a powerful gene editing tool in bridging this gap by allowing researchers to directly target candidate genes in ovarian and metabolic pathways. For instance, experimental models have highlighted the role of *CYP17A1* and *DENND1A.V2* in androgen excess, anti-Müllerian hormone (AMH) in follicular arrest, and insulin receptor substrate 1 (IRS1) and PPARγ in insulin signaling and adipogenesis. To highlight the multifactorial nature of PCOS, animal models, including zebrafish and rodents, have been used to reveal interactions between reproductive and metabolic phenotypes. Nevertheless, most studies remain restricted to single-gene models, and dual-gene models or combined gene editing and hormonal induction models remain underexplored. Future research integrating precision editing, multi-omic platforms, and patient-derived organoids may provide more accurate disease models and novel therapeutic strategies.

## 1. Introduction

Polycystic ovary syndrome (PCOS) is a complex heterogeneous disorder characterized by clinical and biochemical features of androgen excess and disturbances in ovulatory function, primarily affecting females aged 15 to 49 years [1]. Its global prevalence among premenopausal women is estimated at 6–20%, or approximately 1 in 15 females [1]. PCOS represents a leading cause of female infertility and is commonly associated with clinical manifestations such as menstrual irregularities, hirsutism, cystic acne, seborrhea, alopecia and obesity [2]. Beyond reproductive features, the syndrome is often accompanied by metabolic dysfunctions, including insulin resistance, obesity, dyslipidemia, type 2 diabetes mellitus (T2DM) and heightened cardiovascular risk [3]. PCOS has emerged as the primary contributor to anovulatory infertility and, given its high prevalence, imposes significant health and economic burdens on both families and society [3].

From a diagnostic perspective, the Rotterdam criteria remain the principal diagnostic standard, requiring at least two of the following features are present: oligo-ovulation or anovulation, clinical or biochemical hyperandrogenism and polycystic ovaries [1]. Current guidelines recommend characterizing PCOS according to four phenotypes (A–D), which are defined by the presence or absence of the three diagnostic features: hyperandrogenism, ovulatory dysfunction, and polycystic ovarian morphology (PCOM) [4]. Moreover, the phenotypes of PCOS are classified into: phenotype A, meeting all three diagnostic criteria; phenotype B, defined by anovulation and hyperandrogenism; phenotype C, comprising hyperandrogenism with PCOM; and phenotype D, characterized by anovulation with PCOM [5].

PCOS is a complex, multifactorial endocrinopathy that reflects an interplay between genetic predisposition, ovarian dysfunction, neuroendocrine dysregulation, metabolic disturbances and chronic low-grade inflammation [6]. At the endocrine level, PCOS leads to an increased gonadotropin-releasing hormone (GnRH) pulse frequency that preferentially stimulates luteinizing hormone (LH) secretion follicle-stimulating hormone (FSH) secretion [7]—reported in up to 94% of patients [8]. Persistently elevated LH overstimulates ovarian theca cells, upregulating CYP17A1, CYP11A1 and HSD3B2, which enhance androgen synthesis [9,10,11]. Meanwhile, low FSH impairs aromatase (CYP19A1) activity, reducing androgen-to-estrogen conversion and causing follicular arrest [12]. Accumulation of these arrested follicles produces the characteristic polycystic ovarian morphology [1]. Hyperandrogenism, present in over 75% of PCOS cases, leads to follicular atresia, impairs the proliferation of cumulus cells, disrupts angiogenesis, and compromises oocyte maturation [13,14,15,16,17].

Insulin resistance (IR) affects 50–70% of women with PCOS, regardless of body mass index (BMI), and is often accompanied by compensatory hyperinsulinemia [18,19]. Hyperins

Hyperinsulinemia synergizes with LH on theca cells, upregulating CYP17A1 transcription and cytochrome P450 17α-hydroxylase (P450c17α) activity while simultaneously lowering hepatic sex hormone-binding globulin (SHBG) and insulin-like growth factor-binding protein-1 (IGFBP-1) [11,20,21,22,23,24,25,26]. Systematically, IR contributes to the threefold higher prevalence of metabolic syndrome in PCOS, increasing the risk of hypertension, dyslipidemia, obesity and hyperglycemia [27]. Importantly, lifestyle changes or insulin-sensitizing agents such as metformin decrease androgen levels and improve ovulatory function [28,29,30].

Chronic low-grade inflammation is a central feature of PCOS, acting alongside hyperandrogenism and IR [31]. Women with PCOS demonstrate increased C-reactive protein (CRP), interleukin-6 (IL-6), interleukin-18 (IL-18), tumor necrosis factor-α (TNF-α) and white blood cell counts together with dysregulation of pro- and anti-inflammatory mediators in serum and follicular fluid [31,32,33,34,35,36,37]. Elevated cytokines are seen even in lean patients, suggesting inflammation as an intrinsic rather than an obesity-driven factor [38,39,40,41]. Excessive ovarian inflammation reduces oocyte quality, impairs folliculogenesis, and contributes to anovulation [42].

**Figure 1:**
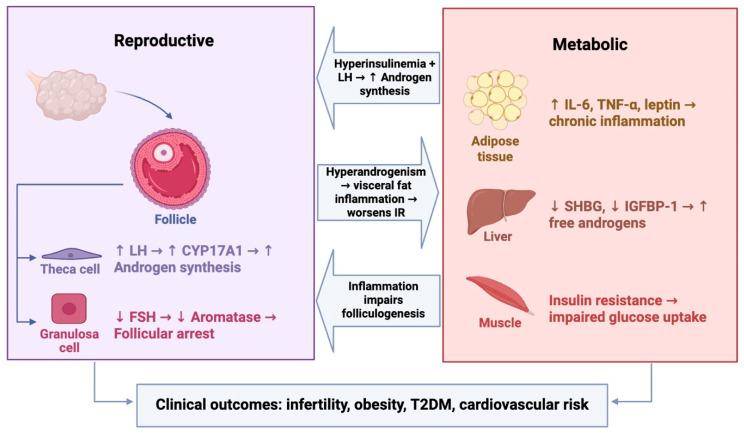
Pathophysiology of PCOS: Reproductive-Metabolic Interactions. LH: Luteinizing hormone; FSH: Follicle-stimulating hormone; IR: Insulin resistance; SHBG: Sex-hormone binding globulin; IGFBP-1: Insulin-like growth factor-binding protein-1; IL-6: Interleukin 6; TNF-α: Tumor necrosis factor alpha. Arrows indicate the directional flow of hormonal, metabolic, and inflammatory signals between tissues, highlighting how hyperandrogenism, insulin resistance, and inflammation interact in a feedback cycle that sustains the reproductive and metabolic abnormalities observed in PCOS.

Hyperandrogenism further induces inflammation in visceral adipose tissue, a key source of IL-6, TNF-α, and leptin, which promote low-grade chronic inflammation, impair insulin signaling, and exacerbate IR [11,43]. Hyperandrogenism also activates nuclear factor kappa B (NF-κB), amplifying inflammatory pathways [44]. Epigenetic regulators, particularly exosomal microRNAs (miRNAs) (e.g., miR-155-5p, miR-222), modulate the phosphatidylinositol 3-kinase/protein kinase B (PI3K/Akt)-GLUT4 pathway and correlate with insulin levels, indicating biomarker potential [45]. This inflammatory state underpins both metabolic complications and increased cardiovascular risk, emphasizing the importance of integrating anti-inflammatory strategies into PCOS management [46,47]. The key reproductive, metabolic, and inflammatory mechanisms underlying PCOS are summarized in Figure 1.

Recent clinical management of PCOS centers on a combination of lifestyle modification and pharmacotherapy to target reproductive and metabolic features [48]. Pharmacological options include combined oral contraceptives and anti-androgens (e.g., spironolactone) to manage hyperandrogenic symptoms, and insulin-sensitizing agents such as metformin which improve insulin resistance and can restore ovulation in some women [48]. For infertility due to anovulation, aromatase inhibition with letrozole has surpassed clomiphene as the preferred first-line ovulation induction agent in many guidelines [49]. Emerging and increasingly studied therapies include incretin-based GLP-1 receptor agonists (e.g., liraglutide, semaglutide) and SGLT2 inhibitors, which have shown promising effects on weight, insulin resistance and some endocrine parameters in short-term trials [50]; inositol is also supported by systematic reviews as a modestly beneficial adjunct for metabolic and ovulatory outcomes in selected patients [51]. While these approaches do not yet constitute disease-modifying therapies for the underlying polygenic and endocrine drivers of PCOS, they provide clinically meaningful improvements in reproductive and metabolic outcomes and are active areas of translational research.

Furthermore, gene editing technology such as clustered regularly interspaced short palindromic repeats (CRISPR)/CRISPR-associated protein 9 (CRISPR/Cas9) will be explored to investigate its potential therapeutic applications. CRISPR/Cas is identified as an adaptive immune mechanism in bacteria and archaeans, which is now being used as gene editing technology [52]. This review aims to provide a comprehensive overview of PCOS, with particular emphasis on the potential use of CRISPR technology in advancing PCOS research.

### Genetic Landscape of PCOS

Over the last decade, genome-wide association studies (GWASs) have significantly advanced our understanding of the genetic underpinnings of PCOS [53]. Studies have identified over 20 susceptibility loci for PCOS across populations, most prominently in *DENND1A*, *THADA*, *LHCGR*, *FSHR*, *INSR*, *YAP1*, *RAB5B* and *HMGA2* [53,54]. These genes converge on pathways critical to gonadotropin signaling, ovarian steroidogenesis, insulin signaling and metabolic regulation [55,56]. *DENND1A*, in particular, has emerged as a pivotal candidate, with the splice isoform *DENND1A.V2* functionally linked to excess androgen production in PCOS theca cells [57,58]. Similarly, *THADA* variants have been associated with impaired energy metabolism, while *INSR* variants implicate abnormal insulin signaling, underscoring the metabolic dimension of PCOS [59,60]. Furthermore, *FSHR* and *LHCGR* polymorphisms highlight dysregulated folliculogenesis and gonadotropin response [53,61,62,63].

Recent studies have expanded the genetic landscape to include *KISS1*, *LEPR*, *SOD2*, *ERBB4*, *WWTR1*, *CHEK2* and *ADIPOQ*, broadening the understanding of PCOS [53]. *KISS1*, which encodes kisspeptin, regulates GnRH secretion and follicular maturation [64,65]. Polymorphisms such as rs4889 have been associated with altered BMI, lower LH and GnRH feedback disruption, with population-level associations in Saudi Arabian women [64,65]. This has functional implications, which include altered kisspeptin neuron morphology in PCOS models and therapeutic exploration of kisspeptin receptor agonists to induce ovulation [66,67,68]. Variants in *LEPR* (rs1137100, rs1137101) affect the binding of leptin and downstream pathways of energy balance and insulin sensitivity [69,70]. Notably, rs1137101 has been shown to have a protective metabolic profile, whereas rs1137100 correlates with increased IR, particularly shown in Bahraini cohorts [69,71]. In addition, *SOD2*, through its *A16V* variant, has been linked to oxidative stress in Chinese women with PCOS, potentially contributing to cellular injury and abnormal gonadotropin ratios [71,72,73,74]. In European and Han Chinese populations, *ERBB4* polymorphisms (e.g., rs113168128) have been reported to be consistently associated with these populations [75,76]. *ERBB4* knockout models in granulosa cells replicate key features of PCOS, such as hyperandrogenism, menstrual cycle disturbances, and metabolic alterations [77]. The Hippo pathway component *WWTR1*, particularly the variant rs144248326, has been associated with infertility and oligomenorrhea [72,78]. Along with *YAP1*, *WWTR1* also regulates IR, and its inhibition enhances the efficacy of metformin [79]. Moreover, *CHEK2* gene variants are involved in regulating ovarian reserve, where rare frameshift/missense mutations may disrupt oocyte apoptosis, delaying reproductive senescence and mirroring the prolonged ovarian reserve seen in PCOS [80,81,82,83]. Modulating adiponectin signaling also contributes to PCOS susceptibility [84]. This has been linked to adiponectin (*ADIPOQ*) and adiponectin receptor 1 (*ADIPOR1*) genes [84,85]. Single nucleotide polymorphisms (SNPs) in *ADIPOQ* show population-specific associations in which six specific SNPs have been reported to have a significant association with the development of PCOS in Bahraini women [86]. However, results seem to differ by ethnicity when looking at *ADIPOQ* gene variants. Specifically, studies in Saudi Arabian and Jordanian women did not show the same association between *ADIPOQ* variants and PCOS as was seen in Bahraini women [87,88]. Additionally, polymorphisms in *ADIPOR1* have been linked to IR in Chinese cohorts [85]. Importantly, in PCOS mouse models, adiponectin treatment ameliorates hyperandrogenism and metabolic dysfunction [89].

Although GWAS has progressed, findings face inherent limitations. Gene prioritization is complicated, as most signals map to non-coding regions [90,91]. Moreover, linkage disequilibrium obscures causality, and effect sizes remain small, accounting for only part of PCOS heritability [55]. In addition, ethnic diversity remains underrepresented, with most data derived from East Asian and European populations [92,93,94]. Furthermore, studies fail to distinguish between the clinical subtypes of PCOS, which may confound results and obscure subtype-specific genetic associations [95]. Finally, the phenotypic heterogeneity in PCOS limits reproducibility between different study cohorts [56]. Functional validation is essential to bridge the gap between association and mechanism. The *DENND1A.V2* model demonstrates this, as it shows a direct causal effect between a GWAS locus and hyperandrogenism [57]. Genome-editing tools such as CRISPR/Cas9 offer powerful strategies to model genetic variants within endocrine and metabolic systems, thereby clarifying their roles in cell signaling and endocrine regulation [96]. Recent advances in genetic and molecular studies are summarized in Table 1, which highlights key genes implicated in PCOS pathophysiology and their potential relevance to CRISPR-based applications.

## 2. Overview of CRISPR Technology and Its Applications in Endocrine Research

CRISPR-based technologies are key tools in endocrine and reproductive research, where precise gene regulation is crucial. The CRISPR/Cas system was adapted from bacterial and archaeal immune defense mechanisms, where it functioned to recognize and cleave the DNA of invading viruses [99]. It was then redesigned as a tool that enabled researchers to modify genetic material in various organisms [100]. Among the various types identified, CRISPR/Cas9 is a type II system that requires a single Cas9 guided by single guide RNA (sgRNA) [101]. The sgRNA directs Cas9 to a genomic target adjacent to a protospacer adjacent motif (PAM) sequence, where the nuclease induces a double-stranded break in the target DNA [100,102,103].

Before the advent of CRISPR/Cas systems, several techniques were used for the manipulation of genetic material. Among these was homologous recombination (HR), a natural process whereby genetic material is exchanged between genomic and exogenous DNA through the crossing over of homologous DNA strands [104]. HR allows for modifications to be introduced to specific genes; however, it is considered to be inaccurate, inefficient and time-consuming [104]. Over time, technological advances led to the development of engineered nucleases, such as zinc finger nucleases (ZFNs) and transcription activator-like effector nucleases (TALENs), which overcame HR [105]. However, ZFNs and TALENs also proved to be problematic due to their complexity, tedious design, high cost and labor-intensive nature [99]. Therefore, the techniques are not suitable for large-scale studies [99]. On the other hand, the CRISPR/Cas system only requires the guide RNA (gRNA) to be modified and constructed, which is faster and easier to design than proteins [99]. In addition, its capacity for multiplexed genome editing, where multiple loci are targeted simultaneously, makes it a more efficient, practical and scalable tool for biomedical research compared to ZFNs and TALENs [106].

These features have made CRISPR particularly valuable, pushing forward reproductive biology by facilitating functional genomics and mutation repair in animal models, and showing promise in the treatment of infertility and genetic diseases [107]. In recent studies, targeted gene editing has allowed researchers to manipulate the activity of hormone receptors, enabling them to understand how these changes affect ovarian function. For instance, Guller et al. applied a CRISPR/dCas9 approach to increase FSHR in human granulosa cells, making them more responsive to FSH and activating important signaling pathways [108]. Most importantly, the study highlights the potential of CRISPR to model and investigate FSH-related conditions, such as PCOS, in vitro [108].

Furthermore, CRISPR has been instrumental in metabolic research by allowing for the precise editing of genes that regulate insulin signaling and glucose metabolism. An estimated 70% of women with PCOS experience IR accompanied by hyperinsulinemia, which increases their risk of developing T2DM and makes these abnormalities a prime candidate for gene-based therapies [109]. In T2DM, IR is characterized by polymorphisms in genes that control insulin signaling pathways, such as IRS1 and IRS2 [110]. Through CRISPR, the function of IRS1 and IRS2 can be enhanced to increase insulin sensitivity in skeletal muscle and adipose tissue, thus improving glucose uptake [110]. Moreover, Matboli et al. have identified a competing endogenous RNA (ceRNA) network of long non-coding RNAs (lncRNAs), miRNAs, and mRNAs linked to IR, along with their target signaling pathways genes [111]. In T2DM, this ceRNA network becomes dysregulated and contributes to impaired insulin signaling [111]. Using CRISPR/Cas9, the researchers knocked out one of the lncRNAs and restored normal expression of the genes linked to T2DM, highlighting a novel approach to the treatment of the disease [111].

These applications demonstrate the versatility of CRISPR as a gene editing tool and its potential for use in conditions such as PCOS, which lies at the intersection between ovarian dysfunction and metabolic disturbance.

## 3. CRISPR in PCOS-Related Ovarian Gene Function

### 3.1. Theca Cell-Specific Genes

Theca cells form the outer layer of developing ovarian follicles and are specialized for androgen biosynthesis [112]. Under LH stimulation, they express steroidogenic enzymes such as CYP17A1 and CYP11A1, which convert cholesterol into androgen precursors later aromatized into estrogens by granulosa cells [113]. In PCOS, theca cell hyperresponsiveness and dysregulated steroidogenesis contribute directly to systemic hyperandrogenism [114,115]. Genetic studies have implicated theca-enriched genes in this phenotype, but functional validation has been limited. CRISPR-based models now offer tools to interrogate these candidate genes with cell-type precision [116].

*DENND1A.V2*, an isoform of the GWAS-nominated *DENND1A* gene, is consistently upregulated in PCOS theca cells and confers a pro-androgenic transcriptomic profile [117]. Overexpression of *DENND1A.V2* in human theca-like cells enhanced *CYP17A1* expression and androgen output, mirroring PCOS-associated hyperandrogenism [57]. While global knockout of *DENND1A* results in embryonic lethality, the potential for CRISPR-based conditional models or humanized knock-ins may allow interrogation of *DENND1A.V2* in vivo [118].

A more direct CRISPR model was developed for CYP17A1, a key enzyme in androgen biosynthesis. Using a doxycycline-inducible CRISPR-Tet-On system, Joseph et al. overexpressed *CYP17A1* specifically in mouse ovarian theca cells [119]. The resultant TC17 females developed marked hyperandrogenism, prolonged estrous cycles and cystic follicle morphology, features that mirror clinical and histologic findings in PCOS [119]. Notably, these defects were reversible upon doxycycline withdrawal, validating this as a temporally controlled, high-fidelity model of androgen-driven PCOS [119].

Furthermore, emerging targets involved in cholesterol trafficking and adrenocortical tropic hormone (ACTH) sensitivity, such as melanocortin 2 receptor (MC2R), steroidogenic acute regulatory protein (StAR) and *CYP11A1*, also exhibit differential expression in theca cells [120,121,122].

### 3.2. Granulosa Cell-Related Genes

Granulosa cells surround the oocyte and mediate follicle growth, estrogen synthesis and ovulatory maturation in response to FSH and LH. In PCOS, granulosa cells often exhibit reduced sensitivity to FSH and dysregulated expression of key factors such as anti-Müllerian hormone (AMH) and its receptor, leading to impaired follicle maturation and ovulatory dysfunction [123,124]. Functional dissection of these pathways using CRISPR-based models has revealed important points of dysregulation in granulosa cell signaling [125].

Recent studies also suggest that A-to-I RNA editing contributes to the pathogenesis of PCOS, particularly through its effects on granulosa cell function [126,127]. In a comprehensive transcriptomic analysis using public datasets from multiple tissues and patient-derived peripheral blood samples, several differentially RNA-edited (DRE) events were consistently identified in PCOS. These events were enriched in genes involved in metabolism, immune regulation, and cell cycle control, including *DEGS1*, *TUBA1B*, *TRIM56*, *STK4*, *ERCC2* and *ZNF83* [126,128,129]. While these editing events have not been fully functionally validated in vivo, their recurrence underscores their potential relevance, and species-specific differences in RNA editing must be considered when extrapolating from animal models.

Functional enrichment analysis of these DRE-associated genes revealed significant involvement in immune and apoptotic pathways, including NF-κB signaling and NOD-like receptor signaling, suggesting a potential role for RNA editing in modulating granulosa cell fate and inflammatory responses in PCOS [37,126,130,131]. These findings are consistent with previously reported dysregulation of PI3K/Akt signaling, oxidative stress responses, and miRNA pathways in granulosa cell apoptosis and abnormal follicular development in PCOS [132,133,134].

RNA-binding proteins (RBPs) may also play a role in regulating these RNA editing events. HNRNPDL, in particular, was predicted to bind multiple DRE sites and is known to be involved in transcriptional regulation and alternative splicing [135,136,137]. Its potential involvement in granulosa cell gene regulation in PCOS represents an important avenue for further study. Among RNA editing enzymes, ADAR has been implicated in multiple biological processes and disease states, but its role in PCOS remains poorly defined [138]. One key ADAR target identified in the study was *EIF2AK2*, which encodes protein kinase R (PKR), a critical component of the innate immune response. *EIF2AK2* was consistently differentially edited in both peripheral blood samples and public PCOS datasets [138,139,140]. This gene is involved in the cellular stress response and antiviral defense by regulating mRNA translation and activating downstream inflammatory pathways. In PCOS, *EIF2AK2* may act through the MAPK signaling cascade, which has been shown to influence granulosa cell apoptosis and steroid biosynthesis [141,142,143]. The EIF2AK2–MAPK axis could represent a mechanistic link between RNA editing and granulosa cell dysfunction in PCOS.

AMH, secreted by granulosa cells of preantral and small antral follicles, plays a central role in restraining follicular recruitment and modulating FSH sensitivity [144]. Both AMH and its receptor, AMHR2, are elevated in PCOS ovaries and are thought to contribute to follicular arrest [145]. A CRISPR-mediated deletion of the GATA-binding site in the mouse *AMH* promoter abolished testicular AMH expression without affecting ovarian AMH, demonstrating sexually dimorphic regulatory mechanisms [146]. Although adult female ovaries remained intact, this model confirmed the feasibility of dissecting *AMH* regulation in vivo [146,147].

A broader approach involving a genome-wide CRISPR knockout screen in sheep granulosa cells cultured with FSH identified over 800 candidate genes implicated in FSH responsiveness [148]. These included multiple epigenetic and transcriptional regulators of FSHR and luteinizing hormone receptor (LHR), expanding the known regulatory landscape beyond canonical FSH signaling and offering a reservoir of candidate genes for future functional studies [148]. In goats (*Capra hircus*), targeted CRISPR knockout of BMPR1B, a receptor involved in the BMP signaling pathway and mutated in high-fecundity sheep (FecB), disrupted granulosa cell proliferation and steroid hormone production [149]. Loss of BMPR1B enhanced SMAD signaling and upregulated FSHR and LHR expression, indicating altered gonadotropin sensitivity [149]. This model recapitulated aspects of the granulosa cell dysfunction observed in PCOS and further established a role for BMP signaling in granulosa cell competence and ovarian physiology. Together, these studies demonstrate that granulosa cell function in PCOS is shaped by complex interactions between hormone signaling, RNA editing and epigenetic regulation. CRISPR-based functional genomics, combined with emerging insights into RNA-level regulation, offer powerful tools to unravel the molecular basis of granulosa cell dysfunction and identify potential therapeutic targets for PCOS.

### 3.3. Folliculogenesis and Ovulatory Dysfunction

Folliculogenesis encompasses the sequential maturation of ovarian follicles from primordial through antral stages, culminating in ovulation [150,151]. This process requires coordinated signaling between granulosa and theca cells, estrogen biosynthesis, and extracellular matrix (ECM) remodeling [151,152]. In PCOS, this tightly regulated sequence is disrupted, leading to follicular arrest and anovulation [153].

CRISPR models targeting genes involved in these pathways have demonstrated many of the structural and functional hallmarks of PCOS ovaries [154]. In zebrafish, CRISPR knockout of *CYP19A1A* abolished estrogen production, resulting in complete ovary-to-testis sex reversal and infertility [155,156]. This extreme phenotype illustrates the essential role of estrogen in establishing and maintaining female reproductive architecture and follicle progression [155,156]. Additionally, CRISPR deletion of *ADAMTS9*, an ECM protease required for follicle rupture, similarly produced a profound anovulatory phenotype [157]. Female *adamts9*^−/−^ zebrafish had underdeveloped ovaries composed entirely of early-stage oocytes and failed to ovulate [157,158]. These results confirm *ADAMTS9* as a key effector of follicle release and oocyte maturation.

To further probe the balance between inhibitory and stimulatory follicular signals, Wu et al. performed a double knockout of *CYP19A1A* and *AMH* [159]. Surprisingly, *AMH* deletion partially rescued the ovarian phenotype of estrogen-deficient zebrafish, enabling some female differentiation and folliculogenesis [159,160]. This highlights the suppressive effect of AMH on follicle recruitment and its potential role in PCOS pathogenesis when elevated.

**Figure 2:**
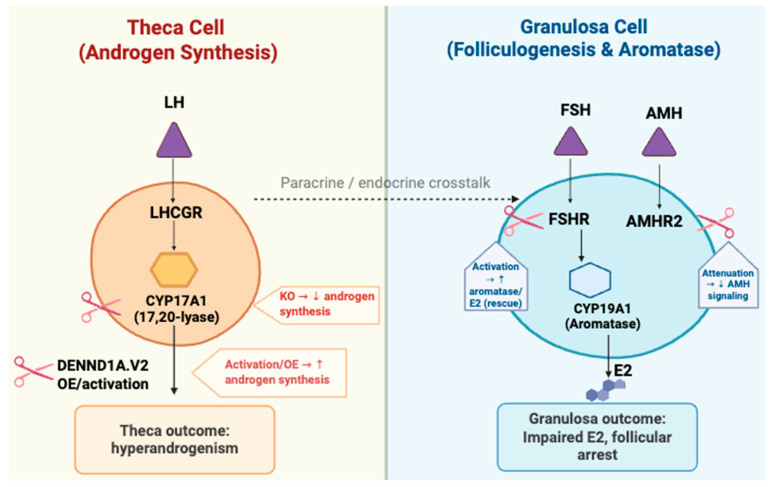
CRISPR targeting of theca and granulosa cell genes in PCOS. LH: Luteinizing hormone; LHCGR: Luteinizing hormone/choriogonadotropin receptor; FSH: Follicle-stimulating hormone; FSHR: Follicle-stimulating hormone receptor; AMH: Anti-Müllerian hormone; AMHR2: Anti-Müllerian hormone type-2 receptor; KO: Knockout; OE: Overexpression. Arrows indicate the direction of hormonal signaling and gene regulation between theca and granulosa cells. Scissors icons represent CRISPR-mediated gene knockout (KO) or activation/overexpression (OE) used to model gene function in PCOS-related pathways.

Finally, the inducible overexpression of *CYP17A1* in theca cells (as described above) provides a mechanistic link between local androgen excess and cyst formation, demonstrating that endocrine cues alone can recapitulate ovulatory dysfunction in the absence of exogenous hormonal induction [115,119,161]. The CRISPR-based gene models described above illustrate the intricate molecular interplay between granulosa and theca cells in ovulatory dysfunction, as summarized in Figure 2.

## 4. CRISPR in Metabolic Gene Function in PCOS

### 4.1. Insulin Signaling Pathways

Using pooled and arrayed CRISPR/Cas9 loss-of-function screens, studies in human adipocyte models have shown that knocking out IRS1 causally impairs insulin signaling, evidenced by the reduced insulin-stimulated AKT2 phosphorylation and glucose uptake. In addition, lipid metabolism is modified, thereby establishing a direct role of IRS1 in cellular IR relevant to PCOS metabolic features [162].

CRISPR-based studies that directly target *INSR* in human adipocytes are limited, and much of our current understanding of *INSR* and *AKT2* in glucose regulation comes from traditional gene-targeted mouse models. For instance, tissue-specific *INSR* knockouts in mice demonstrate that the insulin receptor has an essential role in maintaining glucose homeostasis [163]. Similarly, mice deficient in *AKT2* exhibited hyperglycemia and IR, underscoring AKT2’s crucial role in metabolic control [164]. Likewise, murine embryonic fibroblasts that lack both IRS1 and IRS2 fail to differentiate into adipocytes, showcasing their important role in driving adipogenic transcription factors such as peroxisome proliferator-activated receptor gamma (PPARγ) and C/EBPα [165]. These findings illustrate the mechanistic link between candidate genes and metabolic phenotypes in PCOS, reinforcing their research utility rather than suggesting that gene mutations directly cause the syndrome.

### 4.2. Adipogenesis and Lipid Regulation

**Figure 3:**
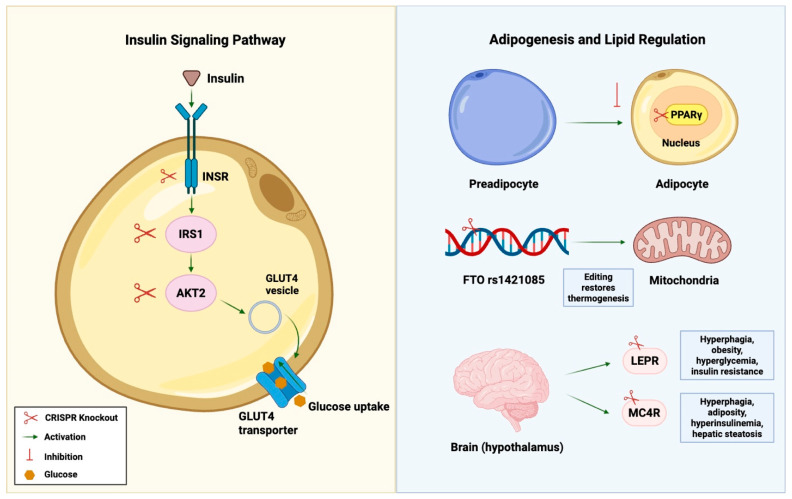
CRISPR knockouts in metabolic pathways of PCOS. GLUT4: Glucose transporter type 4.

In human preadipocytes, CRISPR/Cas9 knockout of PPARγ prevents differentiation into adipocytes, demonstrating the essential role of PPARγ in regulating adipogenesis [166]. In addition to controlling cell lineage, CRISPR/Cas9 editing of the *FTO* rs1421085 variant in primary human preadipocytes restored the program for mitochondrial thermogenesis and reversed the obesity-associated shift toward the differentiation of white adipocytes, thereby establishing a causal link between this common variant and adipocyte functional reprogramming [167]. The application of CRISPR has also clarified neuroendocrine drivers of weight that influence the severity of PCOS. In mice, CRISPR knockouts of the LEPR resulted in hyperphagia, obesity, hyperglycemia, and IR [168]. Likewise, a study by Bao et al. generated LEPR-deficient rats [169]. These knockout rats displayed hyperphagia, obesity, dyslipidemia, glucose intolerance, hyperglycemia, and hyperinsulinemia [169]. This reinforces the evolutionary conservation of LEPR function while offering a robust model to explore PCOS-related metabolic disturbances [169]. A study by Hao et al. investigated CRISPR-mediated knockout of the *MC4R* gene in miniature pigs [170]. Results showed that MC4R-deficient pigs developed marked hyperinsulinemia, IR, hyperorexia, and dysregulated lipid metabolism with substantial hepatic fat accumulation [170]. Moreover, the loss of *MC4R* resulted in hyperphagia and increased adiposity, culminating in hepatic steatosis even in the absence of an atherogenic diet [170]. This reinforces that obesity-linked metabolic circuits mediated through the melanocortin pathway are evolutionarily conserved across species, hence making them relevant to the metabolic burden often seen in PCOS [170]. These metabolic CRISPR models and their effects are illustrated in Figure 3.

## 5. Combined Models: Reproductive and Metabolic Phenotypes

Single-gene models have frequently yielded context-dependent or non-translatable phenotypes that complicate inference for human PCOS. For example, FOXL2 loss in adult mice causes granulosa to sertoli transdifferentiation and ovarian sex-reprogramming; an extreme, species-specific outcome that models ovarian failure rather than the chronic endocrine/metabolic dysregulation of PCOS [171]. Androgen receptor (AR) manipulations produce divergent reproductive and metabolic effects depending on whether AR is knocked out or overexpressed and on the developmental window targeted, underscoring sensitivity to timing and modality [172]. Likewise, oocyte-derived factors such as BMP15/GDF9 show marked species differences. Alleles that alter ovulation rate or fertility in sheep or rodents do not consistently reproduce human PCOS features, limiting cross-species extrapolation [173,174]. These discordant outcomes illustrate why single-gene edits can be misleading and strengthen the rationale for dual/multi-gene, temporally controlled, and cross-species CRISPR approaches to model the polygenic, context-dependent nature of human PCOS.

### 5.1. Dual Gene Models

Dual gene models use CRISPR gene editing to simultaneously target two genes, providing a way to investigate how multiple genetic pathways interact to produce the complex features of a disease [175]. Since PCOS involves both metabolic dysfunction, such as insulin resistance and dyslipidemia, as well as reproductive dysfunction, such as hyperandrogenism and anovulation, dual gene models can offer a more comprehensive insight into the combined effects of these pathways [176]. For instance, CRISPR can be used to edit the reproductive gene *DENND1A*, which is associated with androgen biosynthesis and elevated testosterone levels, while also targeting a metabolic gene, such as *IRS1*, which plays a role in insulin signaling and has been found to be impaired in PCOS patients [177,178]. However, despite this theoretical advantage, there is a gap in the literature, where there are no current studies involving dual gene CRISPR models specific to PCOS. This highlights a significant limitation in the field, as understanding how different genes interact may reveal mechanisms that contribute to the complexity of PCOS.

Due to the absence of dual gene models, most studies have focused on a single-gene editing approach. Interestingly, some of these models displayed both reproductive and metabolic phenotypes. For example, a study by Yan et al. (2017) created gonadal soma-derived factor (GSDF) knockout mutations in zebrafish [179]. The mutant females exhibited PCOS features including infertility, oligo-ovulation, hyperandrogenism, IR, and obesity that mimic PCOS, allowing this study to be used as a model to screen for therapies [179]. While not a dual-gene model, this study demonstrates the effect that a single gene knockout had in triggering both reproductive and metabolic dysfunction, highlighting the potential value of future CRISPR-based dual gene models that target ovarian and metabolic genes simultaneously in investigating the gene-gene interactions that contribute to the pathogenesis of PCOS and providing a more accurate representation of the syndrome.

### 5.2. Integration with Hormonal Induction Models

Traditional animal models of PCOS typically involve induction with letrozole, an aromatase inhibitor, or dehydroepiandrosterone (DHEA) to mimic hyperandrogenism and reproductive dysfunction. Supplementing these protocols with a high-fat diet causes these hormonal models to also develop significant metabolic features, particularly obesity and IR. For instance, a rat model treated with both letrozole and a high-fat diet displayed anovulatory cycles, polycystic ovaries, elevated testosterone, glucose intolerance and dyslipidemia, along with impaired insulin signaling, as evidenced by reduced phosphorylation of *INSR*, *IRS*, *PI3K* and *AKT* in classic insulin-target tissues [180]. Similarly, combining a high-fat diet with DHEA in mice resulted in changes that mimicked those of clinical PCOS. It showed dyslipidemia, elevated LH and serum testosterone, development of multiple ovarian cysts and poor ovarian microenvironment, features that were less pronounced with letrozole or DHEA alone [181].

Although both CRISPR-mediated knockouts of reproductive or metabolic genes and hormone-induced PCOS models (such as letrozole or DHEA induction) are commonly used, a combined approach integrating gene editing with hormonal induction models remains unexplored in the literature. Such combined designs, where CRISPR-modified rodents are further treated with letrozole or DHEA, could provide powerful insight into gene-environment interactions that better replicate the dual metabolic and reproductive phenotypes seen in women with PCOS. Integrating CRISPR gene edits with hormonal induction allows mechanistic exploration of gene-environment interactions, providing insight into human disease pathways without implying immediate clinical application. These models can also further offer a more robust platform for testing therapeutic interventions, as drug responses can be assessed in the presence of both genetic susceptibility and hormonal imbalance, thereby reflecting human disease conditions more accurately. However, to date, no studies have implemented or reported such integrated models.

## 6. Insights from CRISPR Models

### 6.1. Validation of Candidate Gene Roles

While GWAS has successfully identified numerous loci associated with PCOS, these associations alone do not prove that these genes are truly involved in the pathogenesis of the disease [182]. To bridge this gap, CRISPR-based gene editing can directly target the genes, allowing researchers to assess their role in causing the PCOS-relevant phenotypes. For example, CRISPR/Cas9 was used to generate *LNK* (SH2B3) knockout mice, who were fed with a high-fat diet and injected with DHEA to induce PCOS features [183]. Since *LNK* is a crucial regulator of the insulin signaling pathway, the study aimed to investigate its effect on the pathogenesis of PCOS [183]. Compared to wild-type PCOS mice, the *LNK* knockout group exhibited a partially restored estrous cycle and an improved glucose metabolism, indicating that *LNK* may be a target for PCOS clinical treatment [183]. Therefore, the progression from GWAS-only associations to CRISPR-based functional confirmation highlights its potential in identifying genes that play a direct role in PCOS development, helping researchers move from correlation to causation.

### 6.2. Novel Discoveries from Loss-of-Function or Gain-of-Function Studies

Beyond confirming candidate gene roles, CRISPR has also emerged as a discovery tool, uncovering unexpected ways that certain genes behave in PCOS. By enabling targeted loss- or gain-of-function, CRISPR can challenge previous assumptions about a gene’s involvement in PCOS pathophysiology. For example, showing that a gene strongly associated with PCOS in GWAS may have no significant effect in knockout models, while others may unexpectedly drive disease features when overexpressed. Such findings are especially important in a complex condition like PCOS, where the overlapping of reproductive, metabolic, and other pathways provides outcomes that are not always predictable.

For instance, the *THADA* gene encodes thyroid adenoma-associated protein, which is expressed in various tissues, including the ovaries [184]. Studies have revealed its association with disruptions in energy metabolism, increasing susceptibility to obesity and the likelihood of developing PCOS [185]. Additionally, GWASs have identified *THADA* as a candidate gene for PCOS [184]. Therefore, an animal study aimed to prove that *THADA* had an effect in vivo on the female reproductive system, using CRISPR/Cas9 technology to create *THADA* knockout mice [184]. Surprisingly, this study showed that *THADA* ablation did not result in the expected abnormalities, given that the knockout mice had normal fertility, no significant differences in body weight compared to the wild-type mice and no ovarian dysfunction [184]. The study further tested the influence of environmental factors, specifically dietary factors, on PCOS development, and despite stressing the mice with a high-fat-high-sugar (HFHS) diet, they still exhibited a relatively stable ovarian function [184]. This ultimately demonstrates CRISPR’s role in reshaping our understanding of PCOS and shifts our focus to other tissues or pathways.

Recently, a study utilized CRISPR/Cas9 technology to test the involvement of *IGFBP7*, a gene that produces the glycoprotein Insulin-like Growth Factor Binding Protein 7. It is known for regulating vascular remodeling and modulating the pro-angiogenic properties of signaling factors like VEGF-A and IGF [186]. Knowing that *IGFBP7* plays a role in regulating immune dysfunction, its expression levels were tested in PCOS patients and found to be significantly upregulated [187]. Therefore, an animal-based model was created to explore its involvement in immune modulation within the context of PCOS [187]. Briefly, CRISPR was used to create *IGFBP7* knockout mice, and DHEA was administered daily for 21 days to both the knockout and wild-type groups to induce PCOS-like symptoms [187]. As a result, the wild-type mice exhibited features of PCOS, including disruption of their estrous cycle, while the knockout group did not display such features, indicating that turning off the *IGFBP7* gene reversed the disruption caused by the administered DHEA [187]. Furthermore, the ovaries of the *IGFBP7*+/+NC group exhibited cystic follicular enlargement, reduced granulosa cell layers, a great number of immature and atretic follicles and the disappearance of oocytes [187]. Despite being under-researched in the context of PCOS, this study reveals that *IGFBP7* plays a crucial role in the development of PCOS and is worthy of further exploration. Key CRISPR-based loss- and gain-of-function studies investigating PCOS-associated genes are summarized in Table 2.

## 7. Future Directions and Clinical Translation

Although GWASs have uncovered numerous genetic loci associated with PCOS, functional validation of these candidates remains incomplete [188]. CRISPR-based technologies have emerged as powerful tools to interrogate gene function with unprecedented precision, enabling targeted manipulation of PCOS-associated genes across ovarian and metabolic tissues [189]. However, translating these advances into clinically meaningful insights requires overcoming key limitations in current models, namely, species-specific differences in ovarian physiology, limited phenotypic fidelity and insufficient temporal control [190]. To shift from gene identification toward therapeutic translation, future research must prioritize two key strategies: (1) integration of CRISPR with multi-omic platforms and (2) precision gene editing tailored to molecularly defined PCOS subtypes.

### 7.1. Integrating CRISPR with Multi-Omic Frameworks

PCOS is a multifactorial disorder involving gene regulation across ovarian, hypothalamic, and metabolic tissues [45]. Integrating CRISPR perturbations with multi-omics, such as bulk and single-cell RNA-seq, ATAC-seq, ChIP-seq, and proteomics, can capture the multilayered consequences of gene editing in a tissue-specific manner. For example, CRISPR-mediated knockout of *DENND1A.V2* in primary theca cells or steroidogenic cell lines [118] could be coupled with single-cell transcriptomics to identify downstream effectors of androgen synthesis [118,191], while concurrent ATAC-seq could reveal changes in chromatin accessibility at promoters and enhancers governing steroidogenesis [192]. Similarly, base editing of regulatory SNPs within PCOS-associated enhancers (e.g., near *FSHR*, *GATA4* or *LHCGR*) in granulosa-like induced pluripotent stem cell (iPSC)-derived cells can provide direct evidence of variant-driven transcriptional modulation [193,194,195]. In vivo, conditional CRISPR models integrated with spatial transcriptomics or single-cell multi-omic profiling in mouse ovaries could clarify how gene perturbations reshape cellular niches and intercellular signaling during folliculogenesis. These strategies will allow researchers to map the effect of a given gene, as well as its dynamic regulatory network across cell types and time points.

### 7.2. Precision Editing in PCOS

A major barrier to clinical translation is the heterogeneity of PCOS phenotypes [196]. Subgroup-specific genetic dependencies, such as elevated androgen biosynthesis in “hyperandrogenic PCOS” or insulin resistance in “metabolic PCOS,” suggest that therapeutic CRISPR strategies must be personalized [197]. In the near-term, CRISPR-derived tools such as dCas9-fused repressors (CRISPRi) [198,199] or epigenetic silencers (e.g., KRAB, HDACs) can be adapted to transiently modulate genes like *CYP17A1* or *AMHR2* in ovarian cells, without inducing double-strand breaks. This may reduce off-target risks while permitting reversibility, which is ideal for cyclic or temporally restricted pathologies such as anovulation.

Emerging delivery systems such as lipid nanoparticles, viral vectors with tissue-specific promoters, and engineered extracellular vesicles offer potential routes for delivering CRISPR-based therapies to ovaries, liver or adipose tissue [200,201]. For instance, targeted downregulation of *INSR* inhibitors or reactivation of dormant *FSHR* pathways via CRISPRa in granulosa cells could normalize ovulatory cycles in selected patient subtypes [202].

In the long term, integrating patient-specific genomic data with gene editing holds promise for personalized medicine. PCOS organoids and iPSC-derived follicular structures generated from patient cells can serve as preclinical platforms for screening CRISPR-based interventions, toxicity, and efficacy before translation to clinical trials [203,204]. Germline editing remains inappropriate for PCOS applications, but somatic and ex vivo approaches, particularly in reproductive tissues, may enter therapeutic development once safety, efficiency, and reversibility are established [205,206].

### 7.3. Safety, Delivery and Ethical Considerations

Clinical translation of CRISPR for PCOS requires an integrated approach addressing safety, off-target effects, targeted delivery, temporal control, and ethical oversight. Off-target cleavage, large deletions, chromosomal rearrangements, p53-mediated responses, and immune activation are key safety concerns [207,208]. High-fidelity Cas variants (eSpCas9, HiFi Cas9, HypaCas9), base editors, and prime editors significantly reduce these risks by enhancing target specificity and reducing unintended genome modifications [209]. Genome-wide off-target detection methods such as GUIDE-seq, CIRCLE-seq, and Digenome-seq remain essential for preclinical validation [210,211]. Efficient delivery remains critical, with viral vectors (AAV, lentivirus) and non-viral platforms (lipid nanoparticles, polymeric carriers, engineered extracellular vesicles) enabling transient, tissue-specific targeting of ovaries, liver, or adipose tissue while minimizing immunogenicity and genotoxicity [212]. Preclinical evaluation of biodistribution, pharmacokinetics, and large-animal ovarian models is recommended to ensure translational safety [213,214]. Temporal control is particularly important in cyclic ovarian physiology; inducible CRISPR systems, RNP/mRNA delivery, CRISPRi/CRISPRa, and anti-CRISPR proteins allow reversible modulation to prevent long-term adverse effects [215,216]. Ethical and regulatory considerations require balancing permissible somatic applications with prohibitions on germline editing [217,218], while addressing informed consent, intergenerational impact, equitable access, and compliance with national and international guidelines. Long-term monitoring for safety, efficacy, and societal impact is critical, and integrating these strategies with multi-omic and precision editing platforms provides a comprehensive roadmap for responsible CRISPR translation in PCOS research.

## 8. Conclusions

CRISPR technology has provided a turning point in PCOS research, enabling functional validation of candidate genes and clarifying the mechanisms that connect reproductive and metabolic dysfunction. Although most studies still rely on single-gene models, the integration of dual-gene and hormone induction models, patient-derived organoids and multi-omic profiling will likely be necessary to reflect the full complexity of the syndrome. CRISPR not only enhances our understanding of the mechanisms of PCOS, but also sets the foundation for developing precise, gene-targeted therapies.

## Figures and Tables

**Table 1:** Genetic Landscape of PCOS and CRISPR-Relevant Targets.

Gene	Role in PCOS	Potential CRISPR Application
*DENND1A* [57,58]	Overexpressed *DENND1A* isoform leads to excess androgen production	Perform knockout to decrease androgen biosynthesis
*THADA* [59]	Variants are linked to defects in energy metabolism	Model metabolic dysfunction or correct variants to improve insulin sensitivity
*LHCGR* [53,63]	Altered gene expression results in impaired folliculogenesis	Edit variants to restore normal receptor function
*FSHR* [53,62,63]	Reduced receptor sensitivity to FSH leads to defective follicle development and ovulatory dysfunction	CRISPRa to upregulate FSHR expression and increase responsiveness to FSH
*INSR* [60]	Polymorphisms disrupt insulin receptor signaling, which contributes to IR	Target and excise specific *INSR* polymorphisms to restore normal receptor function in metabolic tissues
*YAP1* [97]	Increased activity in variants is associated with disruption in cellular processes and enlarged ovaries	CRISPRi to downregulate *YAP1* overexpression in ovarian granulosa cells
*RAB5B* [98]	Upregulated expression is linked to IR	Correct or knockout *RAB5B* to restore normal insulin signaling
*HMGA2* [54]	Altered expression is associated with hyperinsulinemia and IR	Model *HMGA2* to further understand metabolic effects
*KISS1* [64,65]	Polymorphisms exacerbate ovulatory dysfunction and contribute to abnormal BMI and LH levels	Target SNPs to restore normal GnRH feedback and ovulation
*LEPR* [69]	Variants disrupt leptin binding, impair insulin pathways, and increase IR	Edit variants to improve insulin sensitivity and normalize leptin signaling
*SOD2* [72,73]	*A16V* variant could result in cellular damage and impair gonadotropin balance	Target and edit variant to limit cellular injury and normalize hormone levels
*ERBB4* [77]	Dysfunction can lead to pronounced reproductive and metabolic abnormalities	Create gene knockout models to further investigate how it induces PCOS features
*WWTR1* [79]	Altered expression can contribute to infertility, IR, and influences response to metformin	Inhibit gene to enhance metformin efficacy
*CHEK2* [81,82]	Mutations in *CHEK2* result in irregular oocyte apoptosis and follicular excess	Develop models to better understand how variants contribute to PCOS
*ADIPOQ* [84,86]	*ADIPOQ* polymorphisms are associated with reduced adiponectin levels	Model *ADIPOQ* SNPs to study adiponectin signaling and ethnic-specific effects
*ADIPOR1* [85,89]	Variants impair adiponectin signaling and are linked to IR	Use mouse models to test the effect of treatment on clinical characteristics

FSH: Follicle-stimulating hormone; CRISPRa: CRISPR activation; FSHR: Follicle-stimulating hormone receptor; IR: Insulin resistance; CRISPRi: CRISPR interference; BMI: Body mass index; LH: Luteinizing hormone; SNP: Single-nucleotide polymorphism; GnRH: Gonadotropin-releasing hormone.

**Table 2:** CRISPR Models Applied to PCOS-Relevant Genes.

Target Gene	Model (Species/Cell)	Key Findings	PCOS Relevance	Model Advantages and Limitations
*FSHR* [108]	Human granulosa cells	dCas9 activation increased *FSHR* expression (strongest at 5 min with Gonal-f); estradiol levels also elevated	Demonstrates CRISPR modulation of gonadotropin signaling and steroidogenesis	Human-origin cell model; directly relevant to ovarian physiology. However, the study was conducted in a cell line because primary granulosa cells are difficult to target for epigenome editing.
*IRS1* [162]	Human adipocytes	Loss of function led to insulin-stimulated *AKT2* phosphorylation & glucose uptake; altered lipid metabolism	Links *IRS1* loss to IR & metabolic features of PCOS	Abundant source of adipogenic cells and is suitable for gene editing due to their proliferation ability. The knockout of the effector genes cannot fully replicate the regulatory state.
*PPARG* [166]	Human preadipocytes	Knockout of *PPARG* prevents differentiation of preadipocytes into adipocytes	Shows how *PPARG* plays a role in regulating adipogenesis	Excluding selection markers enhanced knockout efficiency and allowed cells to preserve a high differentiation capacity, but inserting random indels may generate undesired epigenetic changes and result in false positives.
*FTO* (rs1421085) [167]		CRISPR repair of the risk allele restored ARID5B binding, repressed IRX3/IRX5, reactivated browning programs, and increased thermogenesis	Links obesity genetics to PCOS metabolic features	Shows direct manipulation of the FTO risk allele in primary human adipocytes, and highlights its ability to repress thermogenesis in adipocytes, independent of the central nervous system.
*DENND1A.V2* [57]	Human theca-like cells	Overexpression of *DENND1A.V2* enhanced *CYP17A1* expression and androgen output	Results mirror PCOS-associated hyperandrogenism	The model functionally reproduces the hyperandrogenic PCOS phenotype; however, the mechanism by which *DENND1A.V2* regulates *CYP17A1* expression remains unclear. However, only one variant was tested, limiting the scope.
*CYP17A1* [119]	Mouse ovarian theca cells	Overexpression of *CYP17A1* resulted in hyperandrogenism, prolonged estrous cycles, and cystic follicle morphology	Features mirror clinical and histologic findings in PCOS	Ovary-specific overexpression enables temporal control of androgen excess and closely reproduces hyperandrogenic PCOS features. However, systemic metabolic effects were mild, and interspecies differences may limit full translational applicability.
*AMH* [123,124]	Mouse testes and ovaries	CRISPR/Cas9 deletion of GATA-binding site in *AMH* promoter reduced *AMH* expression in fetal/neonate testes but not in adult ovaries	Insights into *AMH* transcriptional control can help explain dysregulated folliculogenesis.	The model demonstrates site-specific control, which facilitates the identification of transcriptional regulatory mechanisms rather than total gene loss. Since the deletion affected fetal/neonatal testes and not adult ovaries, the model cannot fully represent AMH dysregulation throughout life.
*AKT2* [164]	Mice	Knockout mice exhibited growth deficiency, lipoatrophy, IR, hyperglycemia, hyperinsulinemia, and impaired muscle glucose uptake	Demonstrates AKT2’s role in insulin signaling, glucose metabolism, and adipose regulation	Enabled dissection of tissue- and sex-specific *AKT2* functions, particularly in adipose tissue and pancreatic β-cells. The model also mimicked human type 2 diabetes progression, offering translational insight into β-cell compensation and failure. However, the phenotype was strain-dependent, reducing reproducibility across genetic backgrounds.
*LEPR* [168]		Loss of leptin receptor caused hyperphagia, obesity, hyperglycemia, IR, dyslipidemia, and glucose intolerance	Establishes leptin signaling as key to metabolic dysfunction seen in PCOS	The models successfully reproduce the hallmark metabolic disturbances seen in obesity and diabetes, and demonstrate high editing efficiency of CRISPR/Cas9, producing fully penetrant phenotypes without the need for multiple breeding generations. Yet, the line could not be maintained as the mice were severely obese and infertile.
*LNK* (SH2B3) [183]		*LNK* knockout group exhibited a partially restored estrous cycle and an improved glucose metabolism	*LNK* may be a target for PCOS clinical treatment	The model combines genetic (LNK knockout) and hormonal (DHEA + high-fat diet) approaches, replicating both ovarian and metabolic abnormalities. Limitations include the unclear mechanism of FOXO3 (a transcription factor), and hyperandrogenism was underexplored.
*IGFBP7* [187]		*IGFBP7* is linked to cystic follicular enlargement, reduced granulosa cell layers, immature and atretic follicles, and the disappearance of oocytes. Knockout of the *IGFBP7* gene reversed the disruption caused by the administered DHEA	*IGFBP7* plays a role in the development of PCOS	Combines genetic (IGFBP7 knockout) and hormonal (DHEA) models, and uses spleen transcriptomics to uncover immune-endocrine interplay. However, focus was on spleen immune cells, not ovarian tissue.
*CYP19A1A* [155,156]	Zebrafish	Knockout of *CYP19A1A* abolished estrogen production, resulting in complete ovary-to-testis sex reversal and infertility	Highlights the role of estrogen in establishing and maintaining female reproductive architecture and follicle progression	Successful in vivo monitoring of gonadal differentiation. However, an off-targeting effect may occur due to the short recognition site of 18 nucleotides.
*ADAMTS9* [157]		Knockout of *ADAMTS9* led to underdeveloped ovaries composed entirely of early-stage oocytes that failed to ovulate	Demonstrates ADAMTS9’s role in follicle rupture and oocyte maturation	The *ADAMTS9* knockout zebrafish can survive to adulthood, allowing for in vivo study. However, the zebrafish exhibited a strong sex bias towards males, leaving a small number of homozygous knockout females in the offspring of heterozygous crossings.
*GSDF* [179]		Knockout female zebrafish exhibited infertility, oligo-ovulation, hyperandrogenism, IR, and obesity	Metabolic and reproductive phenotypes of PCOS were displayed	The model revealed a clear and reproducible phenotype, where all gsdf mutant fish developed as females, but it lacked rescue experiments to confirm specificity of the phenotype.
*BMPR1B* [149]	Goat granulosa cells	Knockout of *BMPR1B* enhanced SMAD signaling, made cells more sensitive to gonadotropins, reduced cell growth/viability, and removed the usual BMP-4/7 suppression of progesterone production	Shows the gene’s importance in regulating granulosa cell growth and hormone production	The model successfully mimicked the biological phenotype seen in FecB-positive sheep, and used a primary granulosa cell culture, offering a controlled environment to directly assess Smad signaling and steroidogenesis. However, the model does not replicate systemic hormonal interactions, folliculogenesis, or ovulation seen in vivo.
*MC4R* [170]	Pigs	*MC4R*-deficient pigs developed marked hyperinsulinemia, IR, hyperorexia, hyperphagia, increased adiposity, and dysregulated lipid metabolism with substantial hepatic fat accumulation	Highlights melanocortin pathway’s role in obesity-related metabolic dysfunction relevant to PCOS	The model offers a better physiological and metabolic similarity to humans, but failed to assess long-term disease progression.

IR: insulin resistance; ARID5B: AT-rich interactive domain-containing protein 5B; DHEA: dehydroepiandrosterone.

## Data Availability

No new data were created or analyzed in this study.
